# First Outbreak with MRSA in a Danish Neonatal Intensive Care Unit: Risk Factors and Control Procedures

**DOI:** 10.1371/journal.pone.0066904

**Published:** 2013-06-25

**Authors:** Benedicte Grenness Utke Ramsing, Magnus Arpi, Erik Arthur Andersen, Niels Knabe, Dorthe Mogensen, Dorte Buhl, Henrik Westh, Christian Østergaard

**Affiliations:** 1 Department of Pediatrics, Copenhagen University Hospital Glostrup, Glostrup, Denmark; 2 Department of Clinical Microbiology, Copenhagen University Hospital Herlev, Herlev, Denmark; 3 MRSA KnowledgeCenter, Department of Clinical Microbiology, Copenhagen University Hospital Hvidovre, Hvidovre, Denmark; 4 Faculty of Health and Medical Sciences, University of Copenhagen, Copenhagen, Denmark; Amphia Ziekenhuis, The Netherlands

## Abstract

**Introduction:**

The purpose of the study was to describe demographic and clinical characteristics and outbreak handling of a large methicillin-resistant *Staphylococcus aureus* (MRSA) outbreak in a neonatal intensive care unit (NICU) in Denmark June 25^th^–August 8^th^ 2008, and to identify risk factors for MRSA transmission.

**Methods:**

Data were collected retrospectively from medical records and the Danish Neobase database. All MRSA isolates obtained from neonates, relatives and NICU health care workers (HCW) as well as environmental cultures were typed.

**Results:**

During the 46 day outbreak period, 102 neonates were admitted to the two neonatal wards. Ninety-nine neonates were subsequently sampled, and 32 neonates (32%) from 25 families were colonized with MRSA (spa-type t127, SCCmec V, PVL negative). Thirteen family members from 11 of those families (44%) and two of 161 HCWs (1%) were colonized with the same MRSA. No one was infected. Five environmental cultures were MRSA positive. In a multiple logistic regression analysis, nasal Continuous Positive Airway Pressure (nCPAP) treatment (p = 0.006) and Caesarean section (p = 0.016) were independent risk factors for MRSA acquisition, whereas days of exposure to MRSA was a risk factors in the unadjusted analysis (p = 0.04).

**Conclusions:**

MRSA transmission occurs with high frequency in the NICU during hospitalization with unidentified MRSA neonates. Caesarean section and nCPAP treatment were identified as risk factors for MRSA colonization. The MRSA outbreak was controlled through infection control procedures.

## Introduction

Infections due to MRSA have become an increasing clinical problem, causing considerable morbidity and mortality worldwide [1]. Interestingly, some MRSA clones have had the capacity for pandemic spread, while others gave predominantly local epidemics [1], [2]. This globally changing epidemiology has, in part, been caused by community-associated MRSA (CA-MRSA) – new MRSA types initially not found in the hospital and characterized by carrying small SCCmec cassette type IV or V and sometimes the PVL gene [2]. This increase of CA-MRSA has also been seen in Denmark, where the annual number of new MRSA cases has increased significantly from 100 in 2002 to 1293 cases in 2011 [3], though the prevalence of MRSA in *Staphylococcus aureus* bacteraemias still remains low (1.6%–21 of 1293 patients in 2011) [3]. Major health-care associated MRSA outbreaks are rare in Denmark with 22 identified outbreaks in 2011– the largest outbreaks occurred at neonatal departments in the Copenhagen area and Zealand, comprising a total of 26 cases [3]. Furthermore, we have recently shown, in other Copenhagen hospitals, that CA-MRSA has a 9.3 fold lower risk of starting an outbreak than HA-MRSA clones [4]. Interestingly, neonates are highly susceptible for staphylococcal colonization within days of birth, but as they grow older many lose carriage by the age of two [4]–[8]. In some cases colonization will be with MRSA, and MRSA outbreaks in NICU’s have previously been reported in numerous countries (Germany [9], Great Britain [10], [11] Israel [12], [13] Japan [14] Scotland [15], Taiwan [16], USA [17], [18]). Most of these outbreaks have been caused by HA-MRSA, but many of these countries have identified CA-MRSA in the community. Thus the spread of CA-MRSA in their NICU’s may either be introduced by the families or hospital-acquired from HCWs, the NICU environmental, or by inter-hospital transfer of neonates [10], [15], [16], [19], [20]. MRSA outbreaks in NICU’s in countries with low MRSA prevalence are uncommon and have generally been small [21], [22].

When screening for MRSA is used in outbreak control, many neonates are identified as MRSA carriers, but invasive infections have been seen in 14–26% of cases [23], [24].

Attempts have been made to control MRSA in the NICU through treatment of colonized infants, [14], [25], [26]. Poor treatment success has been associated to MRSA colonization of the pharynx [14] and although many NICU use chlorhexidine gluconate body wash, there are safety issues regarding usage in preterm infants and newborns [27].

The purpose of this study was to describe demographic and clinical characteristics and risk factors associated with the first and largest hospital associated (HA)-MRSA outbreak in a NICU in Denmark. This outbreak was caused by introduction of a CA-MRSA.

## Materials and Methods

### The Neonatal Ward

The Neonatal ward at Glostrup Hospital is a level II care centre that receives neonates from three hospitals. The Neonatal ward has two units: The neonatal intensive care unit (NICU) with 20 beds is located on the 6^th^ floor, while the special care baby unit (SCBU) has 10 beds on the 2^nd^ floor. Staffing of these units is by separate nursing teams; but NIC specialists and obstetricians move between neonates. The NICU has a large room for four neonates, with 3 open incubators, facilities for nasal continuous positive airway pressure (nCPAP) and short-term ventilator treatment. A further eight rooms can hold two neonates and five of these rooms can be used for nCPAP. The eight rooms in the SCBU are designed for 10 infants and their mothers.

Neonates from 28 weeks of gestational age and/or with a birth weight of at least 800 grams are admitted to the units. Neonates requiring mechanical ventilation are transferred to a tertiary care centre in Copenhagen, as are infants needing surgical or more complex interventions. Neonates, who have been transferred to the tertiary care centre, will often return when stabilized. NICU neonates are often transferred to the SCBU before being discharged.

The Neonatal ward is open all hours for parents. In the Neonatal ward, the parents are invited to participate in the care of their child under guidance from the staff. Most of the parents take part in tube feeding, bathing and general care of the child. Mothers are supported in early breastfeeding. The parents share a small kitchen with dining facilities and have access to one toilet in each unit.

### The MRSA Outbreak

The outbreak was discovered on July 28^th^ 2008, when MRSA was isolated from a pharyngeal swab from a triplet, who had received prolonged nCPAP. Following MRSA detection, the triplets were isolated and screened for MRSA. The triplets had been transferred from the tertiary care centre NICU in Copenhagen 34 days prior to identification of the outbreak. They were born at the tertiary care centre, and during the first 15 days of hospitalization they were located in a room adjacent to an isolation room, with a neonate isolated due to MRSA spa-type t127. A further 15 neonates were transferred to Glostrup Hospital from the same tertiary care centre NICU during the outbreak period; none of them were MRSA colonized. The triplets were, for this reason, considered to be the index patients at Glostrup Hospital, and their admittance date June 25^th^ marked the beginning of the MRSA exposure period. All 28 neonates hospitalized at the Neonatal ward were isolated and sampled, and the 2 neonatal units were closed for new admittances on August 8^th^, effectively ending the exposure period.

An outbreak management group was established with representatives from the hospital management, pediatric department and daily managers of the two units, a clinical microbiologist and an infection control nurse. The infection control nurse instructed the staff on infection control measures focusing on hand hygiene and hand disinfection, environmental disinfective cleaning and use of protective equipment; gloves and isolation gowns when caring for the neonates. The staff was tested August 5^th^-13^th^ or as soon as possible after those dates. The parents received both written and oral information about the outbreak, hand hygiene and disinfection. All neonates discharged during the exposure period were recalled and screened for MRSA. The remaining NICU neonates were transferred to the SCBU on August 25^th^, and the NICU was temporarily closed, cleaned and disinfected with 3 cycles of vaporised of H_2_O_2_ and silver ions using Sterinis^T^, (Gloster Sante Europe) [28]. The last MRSA colonized neonates were discharged from the SCBU (September 19^th^), 52 days after the index patient was tested positive. The SCBU was then thoroughly cleaned and disinfected with Sterinis^T^. During the outbreak, none of the colonized neonates, family members or HCW developed an infection with MRSA.

### Study Design

All neonatal patients, who had been admitted to the Neonatal ward during the outbreak period, were included in the study. Demographic and clinical data were collected from medical records and from the database, Neobase. Neobase was established in 1995 and registers data from all neonatal wards in Denmark on neonates, while they are hospitalized. Data registered were: date of birth, gender, vaginal delivery or caesarean section, singleton or multiple birth, weight and gestational age at birth, Apgar score at 1 and 5 minutes, asphyxia at birth or chronic lung disease (defined as: Neonate with gestational age (GA) <32 weeks and continued need of oxygen treatment at 36 weeks postmenstrual age), duration of nCPAP treatment, peripheral venous catheter (PVC), antibiotic therapy, dates of admission and discharge or transfer to other hospitals. Discrepancies between the two datasets were thoroughly checked and resolved.

When patients were admitted to the Neonatal ward, the Guardians signed an informed consent that their childs data would be stored in the National database Neobase. This database is in accordance with the rules of the Danish Data Protection Agency and the present study was approved by them (GLO-2009-06). As MRSA is a notifiable disease in Denmark, the study did not require approval from the regional ethical committee but adhered to the ethical guidelines of the hospital. After creation of the research database the data were anonymized.

### Surveillance Cultures

Surveillance swabs from all neonates were obtained from nose, throat, axilla, perineum, and from possible infection sites (urine, skin, eyes). MRSA screening (nose and throat) was performed on all household relatives of MRSA colonized children and on all healthcare workers (HCW) of the Neonatal ward. Swabs were transported in Stuart’s medium (SSI, Copenhagen, Denmark) to the Department of Clinical Microbiology.

### Environmental Cultures

Direct environmental cultures were performed from the room of the last patient with MRSA discharged from the NICU and SBCU, respectively. Cultures were performed before cleaning, after cleaning and disinfection with persulphate (Virkon^T^), and after the subsequent disinfection with the dry mist generator Sterinis^T^. Follow-up cultures were performed after 6 and 15 weeks.

Cultures were performed from 10 locations: alarm bottom, water tap, water handle, alcohol dispenser, bed railing, chair seat, arm rest, laundry cupboard handle, wall and floor. Culture of inanimate surfaces was performed with a 10 cm^2^ staphylococci/Enterobacteriaceae dip slide containing a double agar with neutralizer for detergent and disinfectants (PC2TN) and Baird Parker agar (BV) (Biotrace International, Bridgend, UK). Dip slides were pressed firmly onto the surface, incubated at 35°C and inspected for growth after 1, 2 and 5 days.

### MRSA Identification

Surveillance swabs (from neonates) or a sweep through the colonies from dip slides (environmental cultures) were cultured in a MRSA selective enrichment broth including cefoxitin (Department of Clinical Microbiology, Herlev, Denmark) overnight at 35°C. The following day, 1 µL of the enrichment broth was cultured on chromogenic agar plates (MRSA-ID agar®, BioMeriux) and blood agar plates. *S. aureus* isolates were identified by a positive Staphaurex® test (Remel Europe Ltd., Dartford, UK) and a positive coagulase test (Department of Clinical Microbiology, Herlev). All MRSA isolates were *mec*A positive, Panton-Valentine leukocidin (PVL) gene negative by PCR, *spa* typed by sequencing the staphylococcal protein A gene and SCC*mec* typed as previously described [29], [30]. Antimicrobial susceptibility was performed by disc diffusion for penicillin, erythromycin, clindamycin, gentamicin, vancomycin, linezolid, moxifloxacin, rifampicin and fusidic acid according to SRGA recommendations [31]. An isolate from this outbreak (H597) has been whole genome sequenced (Illumina MiSeq). The potential virulence gene content was identified by a BLAST analysis of the assembled genome against a database of 143 unique DNA sequences designed to detect gene (groups) generally believed to be related to virulence in S. aureus with a selected threshold equal to 90.00% identity within at least 60% of a given gene (Table 1).

**Table 1:** Virulence gene profile of a MRSA isolate from the neonatal outbreak (Mette Theilgaard Christiansen, personal communication).

Virulence factors	Relatedgenes	Nucleotidehomology	Virulence factors	Relatedgenes	Nucleotidehomology	Virulence factors	Relatedgenes	Nucleotidehomology
***Adherence***			***Exoenzyme***			***Toxin***		
**Autolysin**	atl	100.00%	**Cysteine protease**	sspB	100.00%	**Beta hemolysin**	hlb	100.00%
**Cell wall associated fibronectin binding protein**	ebh	93.57%		sspC	99.70%	**Delta hemolysin**	hld	100.00%
**Clumping factor B**	clfB	92.61%	**Hyaluronate lyase**	hysA	100.00%	**Exfoliative toxin type A**	eta	99.47%
**Collagen adhesion**	cna	98.51%	**Lipase**	lip	100.00%	**Exotoxin/superantigen-like proteins**	set2	100.00%
**Extracellular adherence protein/MHC analogous protein**	eap/map	92.92%		geh	100.00%		set3	100.00%
**Fibronectin binding proteins**	fnbA	97.93%	**Serine protease**	splA	100.00%		set4	100.00%
	fnbB	100.00%		splB	100.00%		set16	100.00%
**Intercellular adhesin**	icaR	100.00%		splD	100.00%		set17	100.00%
	icaA	100.00%		splE	100.00%		set18	100.00%
	icaD	100.00%		splF	100.00%		set20	100.00%
	icaB	100.00%	**Serine V8 protease**	sspA	97.78%		set21	100.00%
	icaC	100.00%	**Staphylokinase**	sak	100.00%		set22	100.00%
**Ser-Asp rich fibrinogen-binding proteins**	sdrC	92.14%	**Thermonuclease**	nuc	100.00%		set23	99.86%
	sdrD	96.77%	***Secretion system***				set24	100.00%
	sdrE	100.00%	**Type VII secretion system**	esxA	100.00%		set25	100.00%
	sdrH	97.11%		esaA	100.00%		set26	100.00%
**Staphylococcal protein A**	spa	99.11%		essA	100.00%		hlgA	100.00%
**von Willebrand factor**	vwb	99.93%		esaB	100.00%		hlgC	100.00%
***Host Immune evasion***				essB	100.00%		hlgB	100.00%
**Exoprotein SCIN**	scn	99.72%		essC	100.00%		lukD	100.00%
**Capsule Type 1, 5 and 8**	capA	100.00%		esaC	98.99%		lukE	100.00%
	capB	100.00%		esxB	99.37%			
	capC	100.00%						
	capD	100.00%						
	capP	99.15%						

### Statistical Analysis

All statistical data were generated using SPSS version 16.0 (SPSS Inc. (now IBM)). Data are shown as medians and interquartile ranges. P values for the differences between groups were calculated using Fisher’s Exact Test for categorical variables and Mann-Whitney test for continuous variables. Risk of MRSA colonization were tested in a logistic regression analysis and expressed by odds ratio (OR) estimates. Variables with a P-value of 0.2 or less in the univariate analysis were tested in the multivariate analysis. P<0.05 was considered statistically significant.

## Results

### Patients

**Figure 1:**
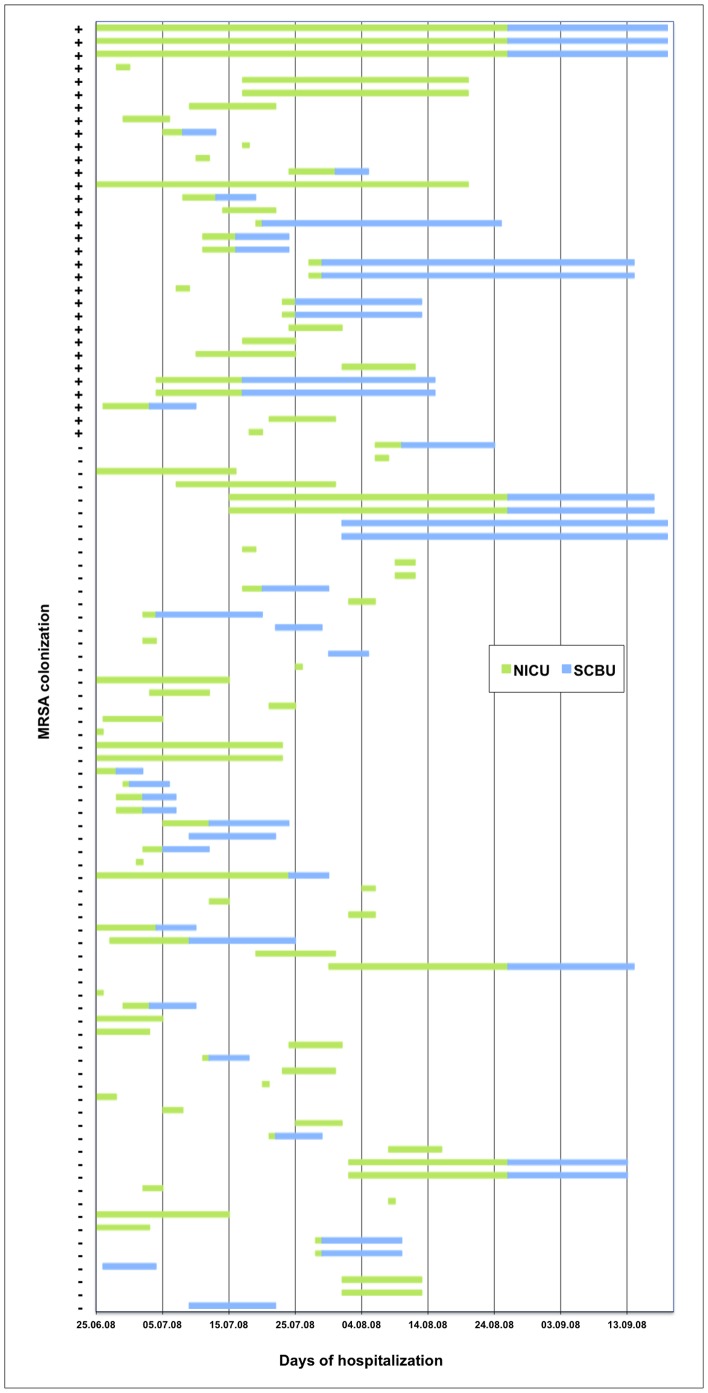
Time course of MRSA outbreak at the 2 neonatal wards. The figure shows the time period of hospitalization for each individual neonate at NICU (green) and SCBU (blue). The first 32 lines (marked with a+sign) are the MRSA colonized neonates and non-colonized neonates are marked with a - sign.

One hundred and two neonates were admitted to the Neonatal ward during the 42-day MRSA exposure period (June 25^th^–August 8^th^). One neonate died due to perinatal asphyxia, and two discharged children could not be contacted. Thus, the study comprised 99 children from 84 families. Figure 1 shows the time period of hospitalization at the two neonatal units and the carriage of MRSA for each individual neonate. Ninety-two neonates were initially admitted to the NICU unit, and 52 were discharged to their homes directly from the NICU (16 colonized with MRSA) and 40 neonates were transferred to the SCBU (16 colonized with MRSA). The first case of transferral to the SCBU occurred on July 2^nd^. Three neonates were readmitted to the NICU from the SCBU. Seven neonates were admitted directly to the SCBU, none of them became colonized with MRSA. Thirty-two percent (32/99) of neonates were colonized with MRSA, comprising 30% of the families (25/84). A higher proportion of colonized neonates were hospitalized at the discovery of the MRSA outbreak (on July 28^th^), compared to discharged neonates (48% (15/31) vs. 25% (17/68), *P = *0.04).

### The MRSA Strain

The MRSA outbreak strain was characterized as *mecA* positive, spa-type t127, Sequence Type ST 1835, a single locus variant of ST1 and PVL negative. The isolates were SCC*mec* V and contained SCC*fus* (Anders Rhod Larsen, personal communication). All isolates were susceptible to erythromycin, clindamycin, gentamicin, vancomycin, linezolid, moxifloxacin, rifampicin, but resistant to fusidic acid. A virulence gene profile is shown in Table 1. The isolate contained Exfoliative toxin type A, however, scalded skin syndrome was not part of the clinical presentation.

### Risk Factors for MRSA Acquisition

Caesarean section and nCPAP therapy were more frequent in neonates colonized with MRSA than in non-colonized children (53% vs. 30%, P = 0.03 and 66% vs. 27%, P<0.001, respectively, Table 2). Neonates colonized with MRSA had been exposed to MRSA for a longer period, than those not colonized (12.5 days vs. 8 days, respectively, *P = *0.04). The risk of being colonized with MRSA increased with 1% per day of the total hospitalization (before and during the triplets admittance); 2.7% per day when exposed to MRSA; 3% per day when treated with nCPAP during the total hospitalization; and 22% per day when treated with nCPAP during the MRSA exposure period.

**Table 2:** Risk factors* for MRSA acquisition.

(No./total) or medians with	Colonized	Not Colonized	Univariate logistic regression		Multivariate logistic regression	
interquartile range	(N = 32)	(N = 67)	OR (95% CI)	p-value	OR (95% CI)	p-value
**Gender (male)**	53% (17/32)	52% (35/67)	1.04 (0.45–2.41)	1		
**Gestational age (days)**	249 (228–266)	262 (226–279)	1.00 (0.98–1.01)	0.27		
**Birth weight (grams)**	2729 (1742–3519)	2490 (1800–3280)	1.00 (1.00–1.00)	0.69		
**Hospital of birth (Glostrup)**	56% (18/32)	54% (36/67)	1.11 (0.47–2.59)	0.83		
**Caesarean section**	53% (17/32)	30% (20/67)	2.66 (1.12–6.35)	0.03	3.74 (1.27–11.0)	0.016
**Twins or triplets**	41% (13/32)	24% (16/67)	2.18 (0.89–5.37)	0.10	1.48 (0.37–5.87)	0.6
**nCPAP treatment**	66% (21/32)	27% (18/67)	5.20 (2.10–12.88)	<0.001	5.88 (1.67–20.7)	0.006
**Transferred from tertiary care centre NICU**	9% (3/32)	23.9% (16/67)	0.33 (0.09–1.23)	0.11	0.07 (0.006–1.27)	0.09
**Chronic lung disease**	6% (2/32)	3% (2/67)	2.17 (0.29–16.12)	0.59		
**Intravascular devices**	59% (19/32)	45% (30/67)	1.80 (0.77–4.24)	0.20	0.89 (0.20–3.95)	0.9
**Treatment with antibiotics**	44% (14/32)	30% (20/67)	1.83 (0.76–4.38)	0.18	1.44 (0.33–6.37)	0.6
**Days hospitalized**	12.5 (7.25–34)	9 (4–27)	1.01 (0.99–1.03)	0.19	0.99 (0.93–1.05)	0.8
**MRSA exposure in days**	12.5 (7.25–34)	8 (4–17)	1.03 (1.00–1.05)	0.04	3.11 (0.40–24.6)#	0.28

* Data with a p-value of 0.2 or less were tested in the multivariate analysis.

# Period of MRSA exposure was logarithmically transformed in the multivariate (Logistic Regression) analysis.

There was no association between MRSA acquisition and gender, gestational age, birth weight, presence of chronic lung disease, PVC or previous treatment with antibiotics (Table 2, *P>*0.05). In the multivariate logistic regression analysis, only treatment with nCPAP and delivery by Caesarean section were independent risk factors for MRSA colonization (*P = *0.006 and *P = *0.016, respectively).

### Family Members and HCW

Among the 25 families with MRSA colonized neonates, two of nine (22%) families with hospitalized neonates had colonized household members; whereas nine out of 16 (56%) families of discharged neonates had colonized household members. In total, 13 family members out of 68 tested (20%) were colonized with MRSA. Two of 161 HCWs (1,2%) were colonized with MRSA.

### Environmental Cultures

In the NICU, MRSA was found on two locations: an alarm button and the floor. In the SBCU, MRSA was found on three locations; a chair seat, a laundry cupboard handle and the floor. Environmental cultures after Sterinis^T^ disinfection of the NICU and SCBU were all without growth of MRSA.

## Discussion

In this large NICU outbreak 32 children from 25 families became colonized with MRSA. The MRSA was introduced into the NICU with the arrival of triplets transferred from the Copenhagen tertiary care NICU. Most likely these triplets have been exposed to the MRSA of a neonate in isolation at this hospital. The MRSA isolates were in all cases the rare spa-type t127, resistant to fucidic acid, described by others as CA-MRSA and previously involved in a CA-MRSA outbreak in the UK [2], [10]. The triplets were in our NICU for 43 days, before MRSA was found in a clinical sample, and during this period 32% of exposed hospitalized infants became colonized, showing the rapid expansion of this clone. Infants still hospitalized at the point of discovery of the MRSA outbreak, compared to infants allready discharged, were more frequently colonized. This was probably due to physical aspects of the ward. The closer to discharge, the further away from the nurses station the infants were placed. In our study the significant risk factors in both univariate and multivariate logistic regression analysis for MRSA colonization were: delivery by caesarean section and nCPAP therapy. We did not find premature birth, low birth weight or multiple gestation as colonization risk factors, although they have been described by others [24], [32], [33]. In MRSA colonized infants, risk factors for MRSA infection have been; gender, gestational age, birth weight, intravascular device, term surgical neonates, multiple gestation, gavage feeding, intubation, [32], [34]–[36]. The MRSA carriage rate in our HCWs was low, 1.2%, but we do not know, if there has been transient carriage. Similar low rates have been found in other countries [12], [19], but MRSA carriage rates of up to 25% have also been described [13], [16]. As found by other investigators environmental contamination with MRSA was an issue, stressing the importance of cleaning [11], [16], [17].

A NICU is a complex hospital ward, regarding infection control procedures, because HCWs and parents are closely involved in the care of the neonates. Although parents are instructed, they are not trained to have good infection control procedures. In our outbreak, Caesarean section and nCPAP were identified as risk factors. Both of these procedures may involve more HCW handling of the neonates at least for the first days after birth. Control of the outbreak was gained through barrier precautions, isolation procedures and intensified disinfective cleaning followed by a final intensive cleaning, when the last MRSA neonate was discharged. The NICU was briefly closed for admittance of new patients. No neonates were treated for MRSA carriage.

Our study was unique in several ways. The MRSA outbreak was very large involving more than one hundred suspected cases, of which 98% were screened. All MRSA belonged to the same CA-MRSA clone, and there is no suspicion of more than one introduction to the NICU. Clinical data collection was complete as all analyzed information could be found in the Neobase and the neonates’ medical records, allowing us to perform multivariate logistic analysis without missing values.

Our study has nevertheless limitations. Only family members with MRSA colonized neonates were screened for MRSA, which could have resulted in unidentified cases.

Also, each individual HCW took their own MRSA surveillance cultures, which could have resulted in sampling failures. However, no swabs were without growth of any microorganisms. Previous studies have shown a greater degree of transmission of MRSA in wards where the nurse staff had an excessive workload [21], [37], but we were not able to include data regarding ratio between staff numbers and neonates in the exposure period due to the retrospective aspect of our investigation.

In conclusion this NICU MRSA outbreak was caused by a CA-MRSA that rapidly spread in a bacterial naive population. Control was established by an outbreak management group that focused on MRSA screening, barrier procedures, isolation, infection control instruction and cleaning. As a consequence of this outbreak all neonates transferred between the four NICUs in Copenhagen are isolated and screened for MRSA on arrival in a new NICU.
